# Comprehensive analysis and expression profiles of the *AP2/ERF* gene family during spring bud break in tea plant (*Camellia sinensis*)

**DOI:** 10.1186/s12870-023-04221-y

**Published:** 2023-04-20

**Authors:** Yujie Liu, Si Chen, Jiedan Chen, Junyu Wang, Mengyuan Wei, Xiaomiao Tian, Liang Chen, Jianqiang Ma

**Affiliations:** Key Laboratory of Biology, Genetics and Breeding of Special Economic Animals and Plants, Ministry of Agriculture and Rural Affairs, Tea Research Institute of the Chinese Academy of Agricultural Sciences, Hangzhou, 310008 China

**Keywords:** Tea plant, AP2/ERF, Spring bud break, Low temperature, Light

## Abstract

**Background:**

AP2/ERF transcription factors (AP2/ERFs) are important regulators of plant physiological and biochemical metabolism. Evidence suggests that AP2/ERFs may be involved in the regulation of bud break in woody perennials. Green tea is economically vital in China, and its production value is significantly affected by the time of spring bud break of tea plant. However, the relationship between AP2/ERFs in tea plant and spring bud break remains largely unknown.

**Results:**

A total of 178 *AP2/ERF* genes (*CsAP2/ERFs*) were identified in the genome of tea plant. Based on the phylogenetic analysis, these genes could be classified into five subfamilies. The analysis of gene duplication events demonstrated that whole genome duplication (WGD) or segmental duplication was the primary way of *CsAP2/ERFs* amplification. According to the result of the Ka/Ks value calculation, purification selection dominated the evolution of *CsAP2/ERFs*. Furthermore, gene composition and structure analyses of *CsAP2/ERFs* indicated that different subfamilies contained a variety of gene structures and conserved motifs, potentially resulting in functional differences among five subfamilies. The promoters of *CsAP2/ERFs* also contained various signal-sensing elements, such as abscisic acid responsive elements, light responsive elements and low temperature responsive elements. The evidence presented here offers a theoretical foundation for the diverse functions of *CsAP2/ERFs*. Additionally, the expressions of *CsAP2/ERFs* during spring bud break of tea plant were analyzed by RNA-seq and grouped into clusters A-F according to their expression patterns. The gene expression changes in clusters A and B were more synchronized with the spring bud break of tea plant. Moreover, several potential correlation genes, such as D-type cyclin genes, were screened out through weighted correlation network analysis (WGCNA). Temperature and light treatment experiments individually identified nine candidate *CsAP2/ERFs* that may be related to the spring bud break of tea plant.

**Conclusions:**

This study provides new evidence for role of the *CsAP2/ERFs* in the spring bud break of tea plant, establishes a theoretical foundation for analyzing the molecular mechanism of the spring bud break of tea plant, and contributes to the improvement of tea cultivars.

**Supplementary Information:**

The online version contains supplementary material available at 10.1186/s12870-023-04221-y.

## Background

The AP2/ERF family is one of the largest transcription factor family that is mainly found in plants [1]. This family detects and binds numerous *cis*-acting elements, such as GCC-box, DRE/CRT, and CEI, and is involved in the expression of various plant genes [2]. In general, AP2/ERFs have at least one conserved AP2 domain, which comprises 60–70 amino acid residues, forming a typical 3D structure of three β-folds and one α-helix [3–5]. According to Sakuma’s classification method [6], the AP2/ERF family can be divided into five categories, namely DREB, ERF, AP2, RAV subfamilies and Soloist.

With the genome-wide identification and analysis of the AP2/ERF family in different plants (such as Arabidopsis [7, 8], rice [7], peanut [9], grapevine [10] and poplar [11]), the research on their functions has been deepened. *AP2/ERFs* are associated with the construction of complex signal transduction pathways in plants. They respond to a variety of stimuli, for example, extreme temperature, drought, high salt and hormones (ethylene, gibberellin and abscisic acid) [2], and have emerged as key regulators of the various physiological and biochemical reactions of plants, which assist plants in effectively improving their ability to cope with adversity stress [12, 13]. Furthermore, *AP2/ERFs* regulate the expressions of target genes during numerous phases of plant growth and development, including cell proliferation and differentiation [14–17], flower growth [3, 18], bud break [19–23] and leaf senescence [24, 25]. Studies in tea plant have shown that the AP2/ERF family contains the most abundant transcription factors in tea plant [26]. Some *AP2/ERFs* have been cloned in tea cultivars ‘Shuchazao’, ‘Anji Baicha’ and ‘Yingshuang’ [27–30]. Further research indicated that these regulators mainly responded to abiotic stress, such as low temperature, high salt and ethylene. RNA-seq analysis was also performed on a short winter dormancy tea cultivar ‘Emei Wenchun’, in which the PB.2659.1, an AP2/ERF transcription factor closest to PtEBB1 in poplar, was screened out by significantly differentially expression analysis [22, 31].

The economic value of tea plant is inextricably connected with its growth and development period. Green tea production accounts for more than 60% of the tea industry in China, with spring elite green tea accounting for more than half of the total output value. However, the economic benefits of spring elite green tea heavily depend on the harvest time. The bud break time in spring has a direct effect on the yield of spring tea since the fresh shoots of tea plant are the main harvest objects. Accordingly, the spring bud break period, as an important agronomic trait of the growth and development period in tea plant, has received extensive attention in the tea industry. A large number of independent genes regulate the release of the bud dormancy and the bud break as a complex process controlled by multiple genes. Genes, such as *CsCDK1* [32], *CsARF1* [33], *CsAIL* (an AP2/ERF transcription factor) [34] and *CsDAM1* [35], have been cloned in tea plant, and their expressions have been confirmed to change during the dormancy release of tea plant. However, the molecular mechanism of tea plant bud break remains largely unknown.

Although some *AP2/ERFs* have been cloned in tea plant, few reports have focused on tea plant bud break. Here, AP2/ERF family in tea plant was analyzed using bioinformatics. The analysis focused on the classification of the gene family, phylogenetic tree information, chromosome localization, gene duplication, *cis*-acting elements of the promoters, gene structure, and conserved motifs. Subsequently, the expression patterns of *CsAP2/ERFs* in different stages of spring bud break were explored by RNA-seq, and the potential interaction genes were mined by weighted correlation network analysis (WGCNA). Finally, temperature and light treatment were conducted on tea plant to explore the response patterns of *CsAP2/ERFs*. This research provided a reference point for further study on the molecular mechanisms of *CsAP2/ERFs* in regulating the bud break of tea plant.

## Results

### Identification of ***CsAP2/ERFs*** in tea plant

According to the annotation information of the AP2 domain (PF00847), 178 *AP2/ERF* genes were identified from the tea plant genome. The protein sequences of these genes were extracted and compared with 147 AP2/ERF proteins in Arabidopsis. Based on the domain characteristics and sequence similarity, the AP2/ERF family in tea plant was divided into five categories, namely DREB subfamily (52 members), ERF subfamily (88 members), AP2 subfamily (30 members), RAV subfamily (4 members) and Soloist (4 members). We named these genes in accordance with the family classification and genome location information, and recorded them in Table S1. Thereafter, the basic physicochemical properties of *CsAP2/ERFs* were analyzed. The amino acid lengths of *CsAP2/ERFs* ranged from 67 aa (*CsSoloists-01*) to 748 aa (*CsAP2-29*), and the protein molecular weight (MW) ranged from 74.54 kDa (*CsSoloists-01*) to 811.66 kDa (*CsAP2-29*). The theoretical isoelectric point (pI) varied from 4.49 (*CsERF-36*) to 10.24 (*CsERF-70*).

### Phylogenetic analysis of ***CsAP2/ERFs***

**Fig. 1:**
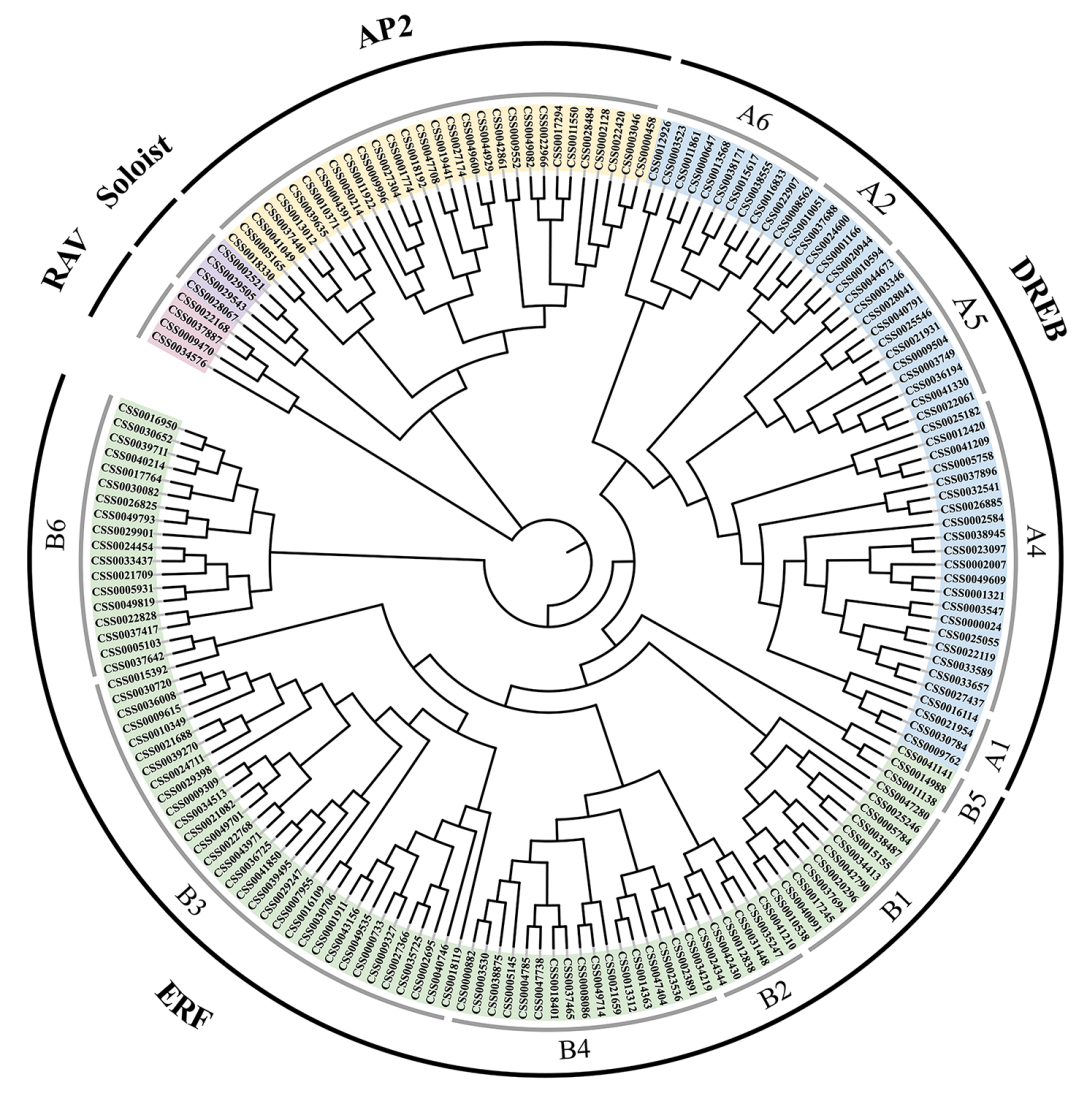
A neighbor-joining phylogenetic tree of the AP2/ERF family in tea plant. The phylogenetic tree was constructed based on 178 conserved domain sequences of CsAP2/ERFs. The CsAP2/ERF family was allocated to five subfamilies (DREB subfamily with groups A1-A6, ERF subfamily with groups B1-B6, AP2 subfamily, RAV subfamily and Soloist), and covered with different colors

An unrooted phylogenetic tree was constructed in tea plant by using the conserved sequences of proteins in the CsAP2/ERF family (Fig. 1). Meanwhile, the DREB and ERF subfamilies were subdivided into six groups based on two main distribution methods proposed by Sakuma [6] and Nakano [7]. Table 1 summarizes the number of AP2/ERFs of tea plant, Arabidopsis [7], poplar [11] and grapevine [10]. Overall, the ERF subfamily has a numerical advantage in all species listed. According to the comparison of the distribution of subfamilies in four species, the distribution of CsAP2/ERFs was more similar to that of poplar and grapevine. Moreover, the A3 group (IVb) of DREB subfamily was missing in tea plant and grapevine, which is consistent with previous reports [10, 26].

**Table 1:** Summary of the AP2/ERF family in tea plant, Arabidopsis, poplar and grapevine

Distribution method	Sakuma’s method	Nakano’s method	*Camellia sinensis*	*Arabidopsis thaliana*	*Populus trichocarpa*	*Vitis vinifera*
Family/subfamily	Group	Group				
DREB	A1	IIIc	4	6	6	7
	A2	IVa. IVb	6	8	18	4
	A3	IVb	0	1	2	0
	A4	IIIa, IIIb, IIId, IIIe	20	16	26	13
	A5	IIa, IIb, IIc, IIIa	11	16	14	7
	A6	Ia, Ib	11	10	11	5
Total			52	57	77	36
ERF	B1	VIIIa, VIIIb	12	15	19	7
	B2	VII	6	5	6	3
	B3	IXa, IXb, IXc,Xb	32	17	35	37
	B4	Xa, Xc	17	8	7	4
	B5	VI	3	8	8	4
	B6	Va, Vb, VI-L, Xb-L	18	12	16	18
Total			88	65	91	73
AP2			30	18	26	18
RAV			4	6	5	4
Soloist			4	1	1	1
Total			178	147	200	132

### Gene location and genome synteny of ***CsAP2/ERFs***

**Fig. 2:**
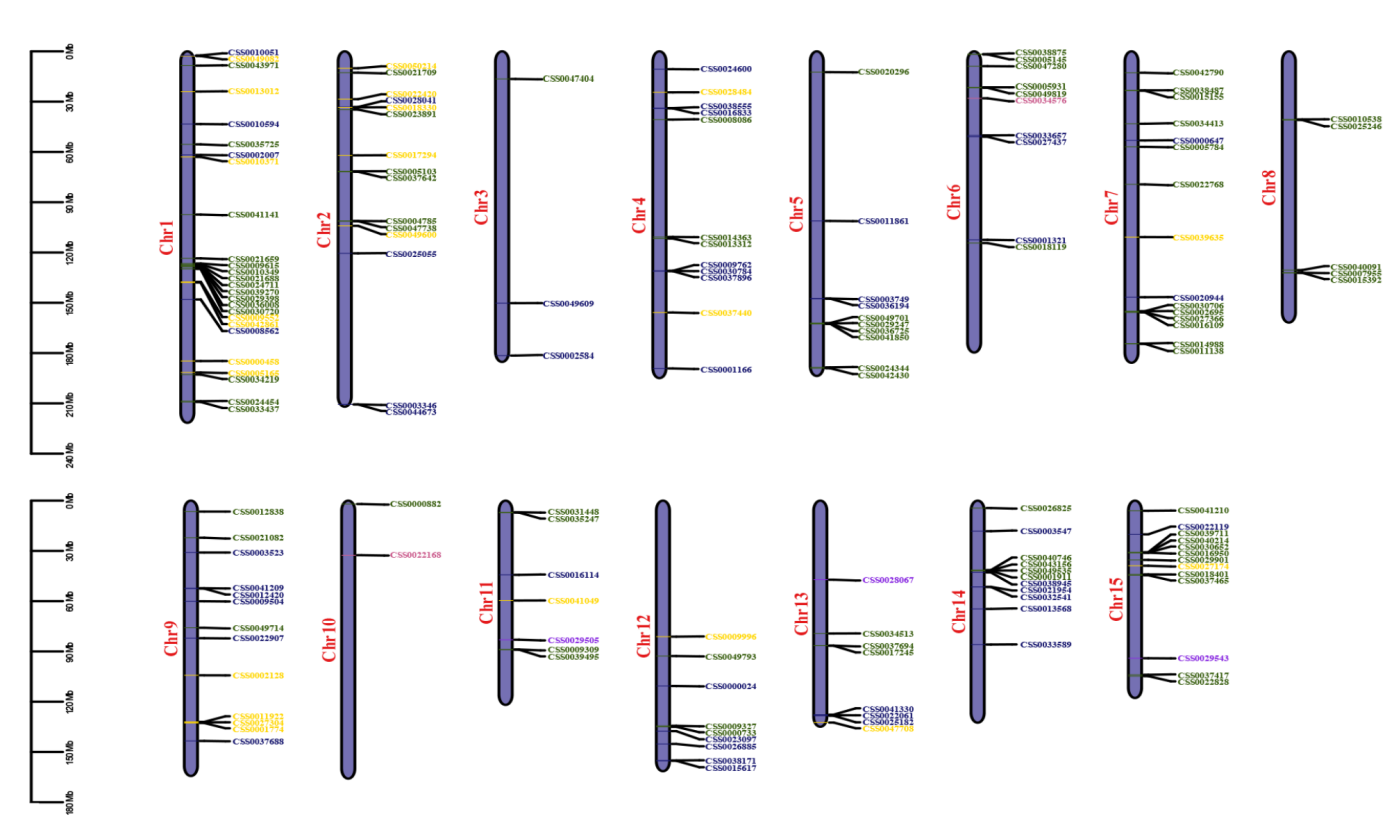
Genome location of 159 *CsAP2/ERFs* on 15 chromosomes. The chromosomal positions of *CsAP2/ERFs* were mapped according to the genome of tea cultivar ‘Shuchazao’. The subfamilies were shown in different colors (*CsDREBs* in blue, *CsERFs* in green, *CsAP2s* in yellow, *CsRAVs* in pink and *CsSoloists* in purple)

159 *CsAP2/ERFs* were unevenly distributed across 15 chromosomes, except for 19 genes on contigs (Fig. 2). Chr1 had the largest number of *CsAP2/ERFs* (26 genes), whereas Chr10 had the smallest (2 genes). *CsDREBs* located on every chromosome except Chr 8 and Chr 10. Apart from that, two *CsRAVs* were mapped on Chr6 and Chr10, as well as three *CsSoloists* were mapped on Chr 11, Chr 13 and Chr15. *CsERFs* were more likely to get clustered on chromosomes compared with other *CsAP2/ERFs*. Clustered *CsERFs* were easy to spot in Chr1, Chr5, Chr7, Chr14 and Chr15. Every chromosome contained more than two types of *CsAP2/ERFs*, while Chr 8 only has *CsERFs*.

**Fig. 3:**
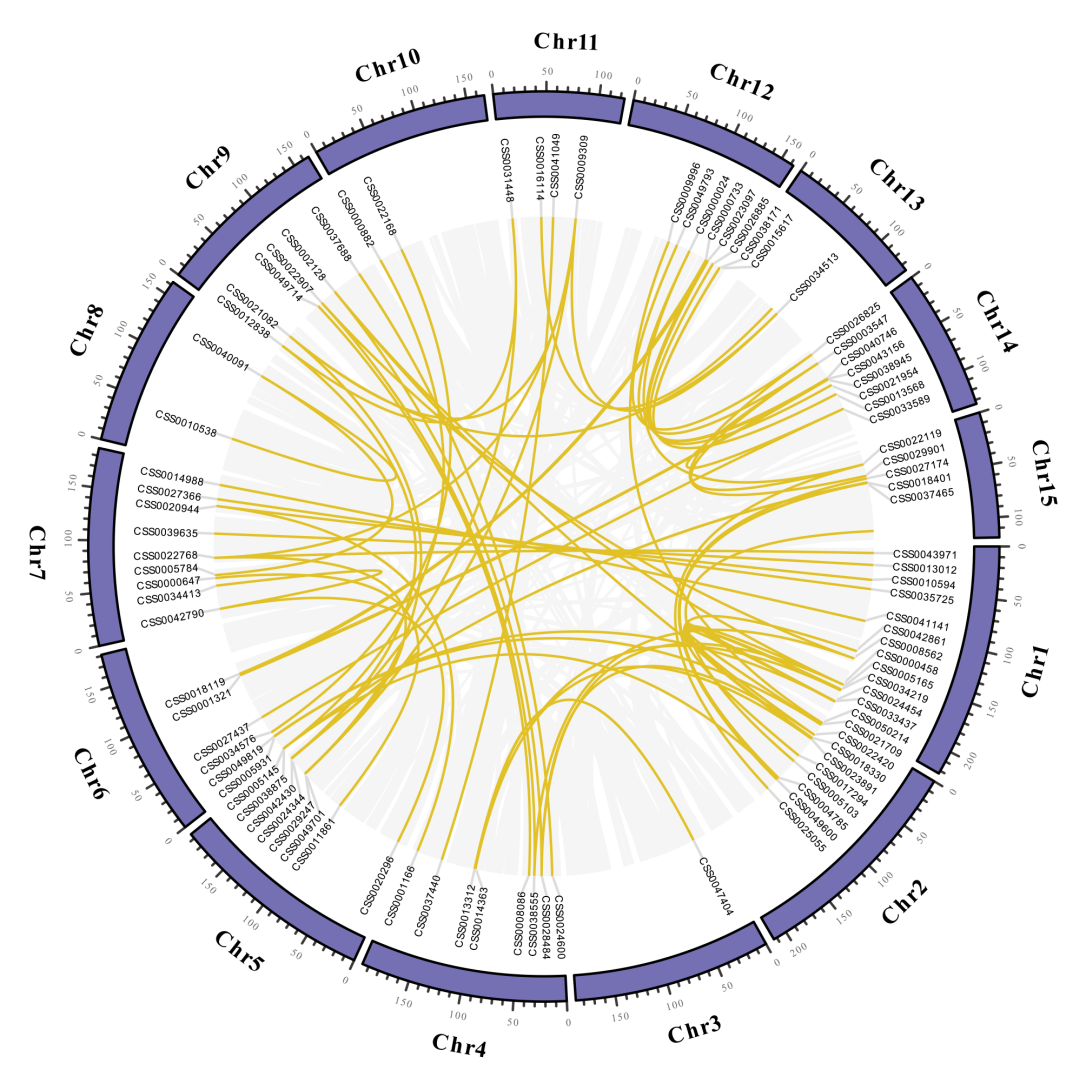
The synteny analysis of *CsAP2/ERFs*. The value on each chromosome represents the chromosome length in Mega base (Mb). The gray lines indicate all synteny blocks in the genome of tea cultivar ‘Shuchazao’, and the gold lines denote the whole genome duplication (WGD) or segmental duplicated gene pairs of *CsAP2/ERFs*

**Fig. 4:**
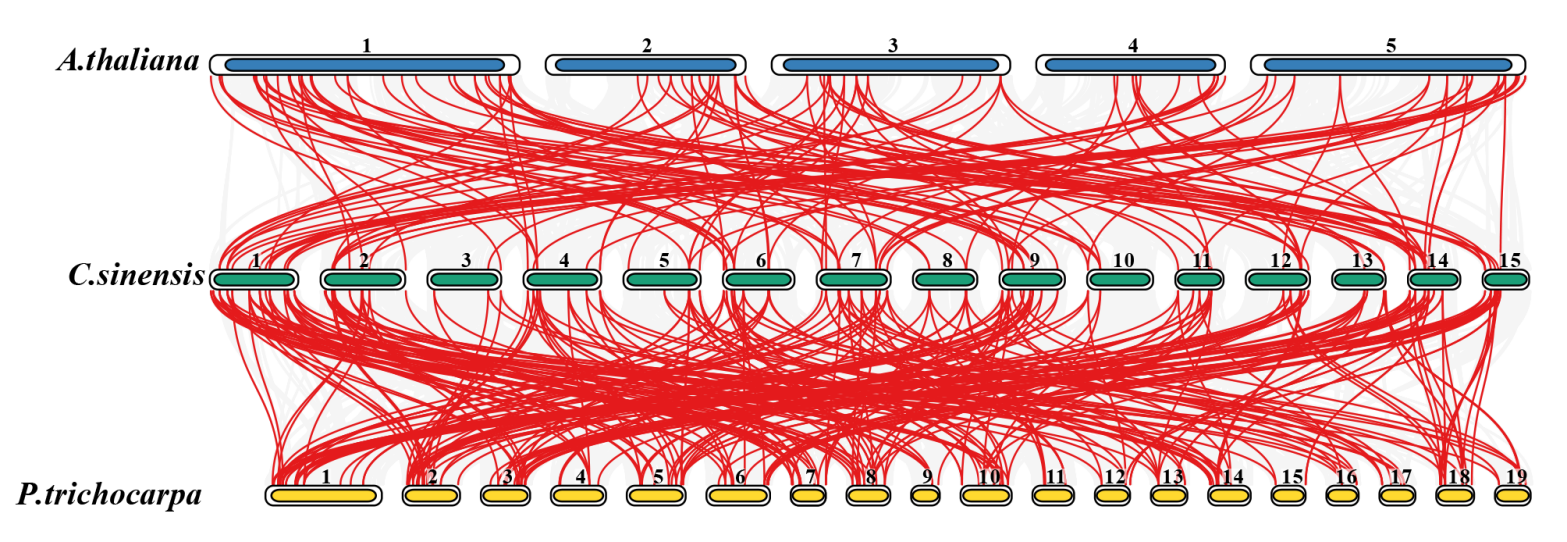
The synteny analysis of *AP2/ERFs* between Arabidopsis, tea plant and poplar. Gray lines indicate all synteny blocks between tea plant and the other two species. The red lines indicate the orthologous *AP2/ERFs*

We detected gene duplication events in the *CsAP2/ERF* family in tea plant (Fig. 3), and 72 pairs of whole genome duplication (WGD) or segmental duplication events and 13 pairs of tandem duplication events were found. Thus, WGD or segmental duplication was the main expansion pattern of the *CsAP2/ERF* family. Meanwhile, tandem duplication promoted the extension of *CsDREBs* and *CsERFs*. We calculated the Ka/Ks values for all paralogous genes to assess the selection pressures (Table S4). The results showed that the ratios of Ka/Ks between all paralogous gene pairs were less than one, indicating that purifying selection was dominant during the evolution of the *CsAP2/ERFs*. To further explore the potential evolutionary mechanisms of the *CsAP2/ERF* family, the synteny analysis of the *AP2/ERF* families of tea plant, Arabidopsis and poplar was conducted (Fig. 4). The synteny relationships are presented in Table S5. The results showed that tea plant has more *AP2/ERF* gene pairs with poplar (300 pairs) than with Arabidopsis (133 pairs). This result suggested that the *AP2/ERF* family of tea plant was evolutionarily similar to poplar.

### Gene structure and conserved motif analysis of ***CsAP2/ERFs***

The CDS, UTR and introns were analyzed to characterize the gene structure of *CsAP2/ERFs*. The *CsAP2s* had the unique gene structures in the *CsAP2/ERF* family, and they tended to include several short tandem CDS regions (Figure S2). Comparatively, the other four subfamilies had fewer number of CDS, ranging from one to four in the majority (Figure S3-S6), and CSS0012420 (*CsDREB*) was one exception which had seven CDS. All *CsRAVs* and several *CsDREBs* had a long, complete CDS almost covering the whole genes. A total of 76 *CsAP2/ERFs* had the UTRs in their structure, but nine of them only had the 5’-UTR as well as 16 only had the 3’-UTR.

15 conserved motifs were predicted by the MEME to investigate the key motif in *CsAP2/ERFs*. The motif compositions were distinct in different subfamilies (Figure S2-S6). However, motif 1 was conserved in all *CsAP2/ERFs* except all *CsSoloists* and two *CsERFs* (CSS0005103 and CSS0037642). *CsAP2s* had two main composite patterns, one was motif 1-motif 3-motif 1, and the other was motif 7-motif 10-motif 2-motif 1. Based on these basic patterns, the composition of *CsAP2s* would add or replace some motifs. *CsDREBs*, *CsERFs* and *CsRAVs* had a similar motif composition, a series connection of motif 2-motif 4-motif 1. On this basis, more than half of *CsDREBs* added motif 6, and only one *CsDREB* (CSS0033589) added motif 8. Motif 11 was conserved in *CsRAVs* compared with motif 9. Different from *CsDREBs* and *CsRAVs*, the motifs of *CsERFs* were more diverse in groups B3 and B6. Motif 9 was found in group B6, while motifs 8, 13 and 14 were found in group B3. Besides, *CsSoloists* were half more covered with motif 12.

### Putative ***cis***-acting element analysis of ***CsAP2/ERFs***

The PlantCARE database was exploited to analyze the *cis*-acting elements in *CsAP2/ERFs*. As the results showed in Table S6, the elements were classified into five categories: hormone response, plant growth and metabolic regulation, stress response, structural elements and transcription factor binding sites. The hormone responsive elements include five types: abscisic acid response (ABRE), auxin response (TGA-element and AuxRR-core), gibberellin response (TATC-box, P-box and GARE-motif), MeJA response (TGACG-motif and CGTCA-motif) and salicylic acid response (TCA-element). Plant growth and metabolic regulation elements contain MSA-like (cell cycle regulation), circadian (circadian control), HD-zip 1 (differentiation of the palisade mesophyll cells), ACE (light response), CAT-box (meristem expression), RY-element (seed-specific regulation) and so on. The third type is stress responsive elements, such as the wound responsive element (WUN-motif) and the low-temperature responsive element (LTR). The fourth type consists of structural elements, such as the protein binding site (Box III/HD-Zip 3) and promoter and enhancer regions (CAAT-box). Finally, the common transcription factor binding sites include the MYB binding site (MBS, MBSI and MRE) and the MYBHv1 binding site (CCAAT-box).

In *CsAP2/ERFs*, the most widely distributed *cis*-acting elements are the structural elements, which account for more than 70% of the total amount in the five subfamilies, and are as high as 80.34% in *CsSoloists*. In addition, plant growth and metabolic regulation elements account for more than 10% in each subfamily, the highest is 13.98% in *CsERFs*, followed by 13.20% in *CsAP2s* and 12.72% in *CsRAVs*. The distribution of the hormone responsive elements widely varied, ranging from 8.67% (*CsRAVs*) to 3.42% (*CsSoloists*). *CsDREBs* (6.31%) and *CsERFs* (6.51%) have similar numbers of hormone responsive elements, while *CsSoloists* (3.42%) have slightly less. Stress responsive elements accounted for 3.13% (*CsDREBs*), 3.14% (*CsERFs*), 4.53% (*CsAP2s*), 2.89% (*CsRAVs*), and 4.56% (*CsSoloists*) of the total, respectively. Among the five subfamilies, transcription factor binding sites are the least distributed, occupying only about 1% of the total *cis*-acting elements.

### Expression profiles of ***CsAP2/ERFs*** during spring bud break

**Fig. 5:**
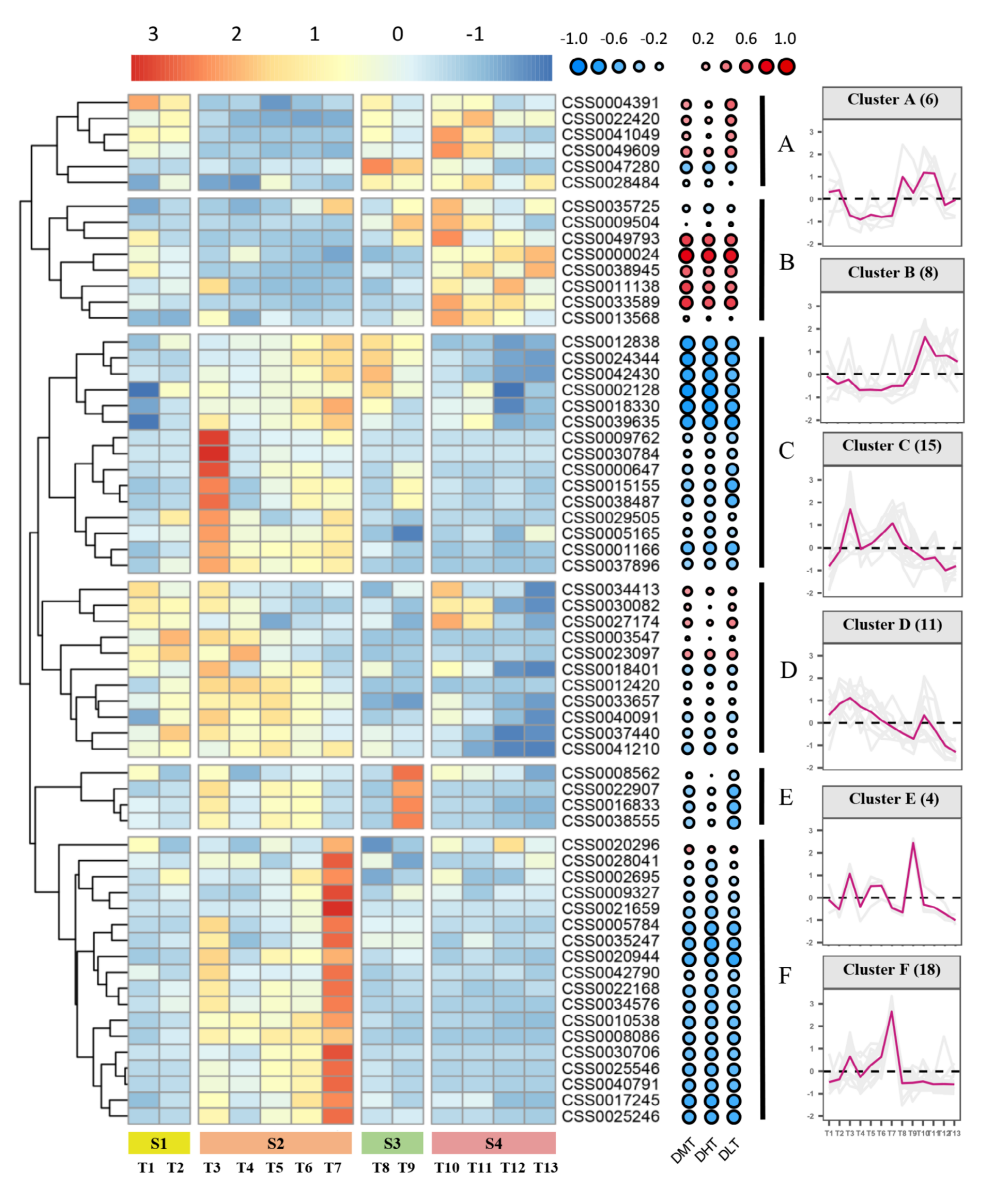
Heatmap of *CsAP2/ERFs* during the different stages of tea plant bud break. The 62 *CsAP2/ERFs* clustered into six groups based on their specific expressions during the four stages (S1-S4) of tea plant bud break (S1: T1-T2, S2: T3-T7, S3: T8-T9 and S4: T10-T13). The circular heatmap showed the correlation analysis between the environmental factors and gene expression levels (DMT: daily mean temperature, DHT: daily maximum temperature, DLT: daily minimum temperature). The graph on the right of the heatmap showed the expression patterns of the six distinct clusters. Gene expression levels were represented by standardized FPKM values. The standardization method was z-score, z = (x − µ)/σ (x: original value, z: transformed value, µ: mean and σ: standard deviation)

The expression profiles of *CsAP2/ERFs* were detected by RNA-seq, and the results were analyzed from T1 (November 1, 2021) to T13 (March 19, 2022) to clarify the role of *CsAP2/ERFs* during tea plant bud break, which were classified into four stages (S1: paradormancy, S2: endodormancy, S3: ecodormancy and S4: bud expansion and break) [36–38]. A total of 62 *CsAP2/ERFs* were selected by the FPKM values (FPKM > 5) (Table S7). These genes were hierarchically clustered according to the expression similarities and grouped into six expression modules, naming clusters A-F for further analysis (Fig. 5). Cluster F contained the largest number of *CsAP2/ERFs* (18 members). 15 genes belonged to cluster C, followed by clusters D, B and A, which contained 11, 8 and 6 *CsAP2/ERFs* severally. Besides, cluster E contained the last number of *CsAP2/ERFs* (4 members).

The analysis of the clustering results indicated that there were two main expression patterns. Clusters A and B more actively expressed in the stages close to bud break (S3 and S4), while other clusters in the early stages (S1 and S2). The expression of cluster A sharply decreased after S1, and it was almost not expressed in the whole S2. The expression of cluster B was similar to that of cluster A in this phase. The expression recovery of clusters A and B was observed in S3 and slightly declined in S4. In contrast, other clusters were inactive in both S3 and S4 periods except for cluster E with a transient recovery of expression in T9 (S3). Clusters C, E and F showed apparent expression peaks during the whole expression process compared with clusters A, B and D. The highest expression levels were evident in T3 (S2), T9 (S3) and T7 (S2). The expression peak of cluster E appeared at T9, and two obvious fluctuations occurred before this. Clusters C and F had virtually identical expression patterns, and they showed high expression levels at T3 and T7. In contrast with cluster C, the expression peak of T7 was higher than that of T3 in cluster F.

### Expression profiles of the potential interacting genes of ***CsAP2/ERFs***

**Fig. 6:**
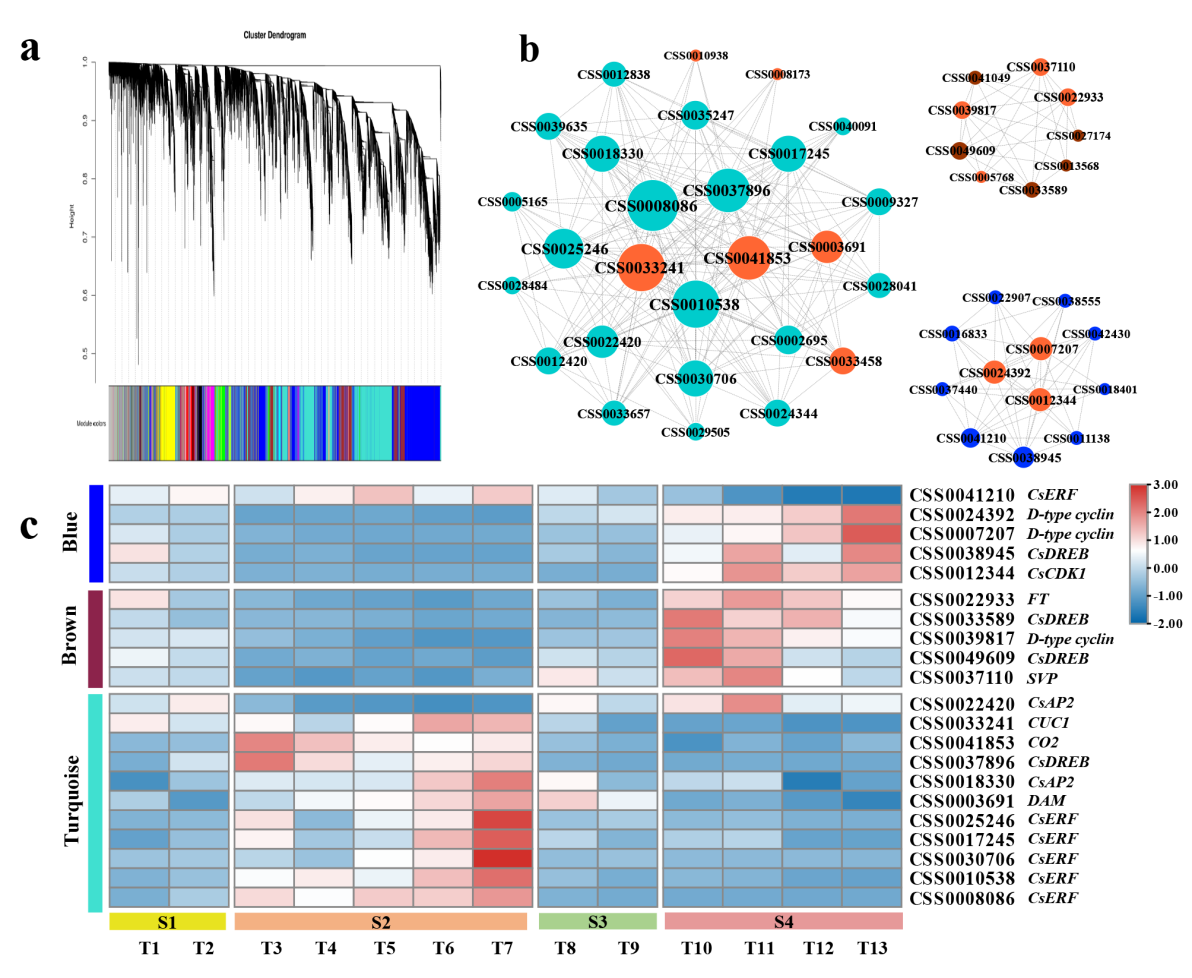
Screening of potential interacting genes based on WGCNA. (**a**) Clustering dendrogram of the average network adjacency for identifying potential interacting genes. The genes in modules are marked with different colors. (**b**) Gene networks in blue, brown and turquoise modules. The *CsAP2/ERFs* in these three modules are severally colored with the module color, and the orange dots show the bud break related genes. The dot size represents the degree values. (**c**) The expression profiles of the selected genes from the blue, brown and turquoise modules

WGCNA was performed to explore the potential interaction genes of *CsAP2/ERFs* to further elucidate the mechanism of *CsAP2/ERFs* in tea plant bud break regulation (Fig. 6a). In the clustering module of WGCNA, the previously mentioned *CsAP2/ERFs* (picked by FPKM > 5) were mainly divided into three modules, namely, Blue, Brown and Turquoise. Meanwhile, some reported genes involved in the bud break of woody perennials appeared in these three modules. The co-expression network between *CsAP2/ERFs* and bud break related genes were analyzed (Fig. 6b). The top 50% genes in each network were selected according to the degree values to further analysis. Subsequently, referring to the correlation coefficients (Table S8), the expression profiles of 12 *CsAP2/ERFs* and nine highly related genes (|r| > 0.70) were shown in Fig. 6c.

CSS0041210 and CSS0038945 was classified into Module Blue. Three cyclin-related genes (CSS0024392, CSS0007207 and CSS0012344) were found in this module. These genes had similar expression patterns with CSS0038945 (r > 0.80) and were negatively correlated with the expression of CSS0041210 (r ≤ −0.85). The expressions of genes listed in Module Brown were consistent (r > 0.70), and their expression peaks appeared at S4 and decreased with the development of the tea buds. The gene expression peak in Module Turquoise mainly appeared in S2, while CSS0022420 was not active in this phase. The results of the correlation analysis showed that the expression of CSS0022420 was negatively correlated with CSS0041853 (*CO2*), and the correlation coefficient was −0.73. Concurrently, CSS0041853 (*CO2*) was positively correlated with the expressions of CSS0010538, CSS0008086 and CSS0037896, and the correlation coefficients were 0.78, 0.86 and 0.95, respectively. The expression of CSS0010538 was also highly consistent with CSS0003691 (*DAM*) and CSS0033241(*CUC1*), with the correlation coefficients of 0.70 and 0.79 separately. CSS0033241(*CUC1*) had the highest expression correlation with CSS0008086 (r = 0.80).

### Expression profiles of ***CsAP2/ERFs*** under low and high temperature treatment

**Fig. 7:**
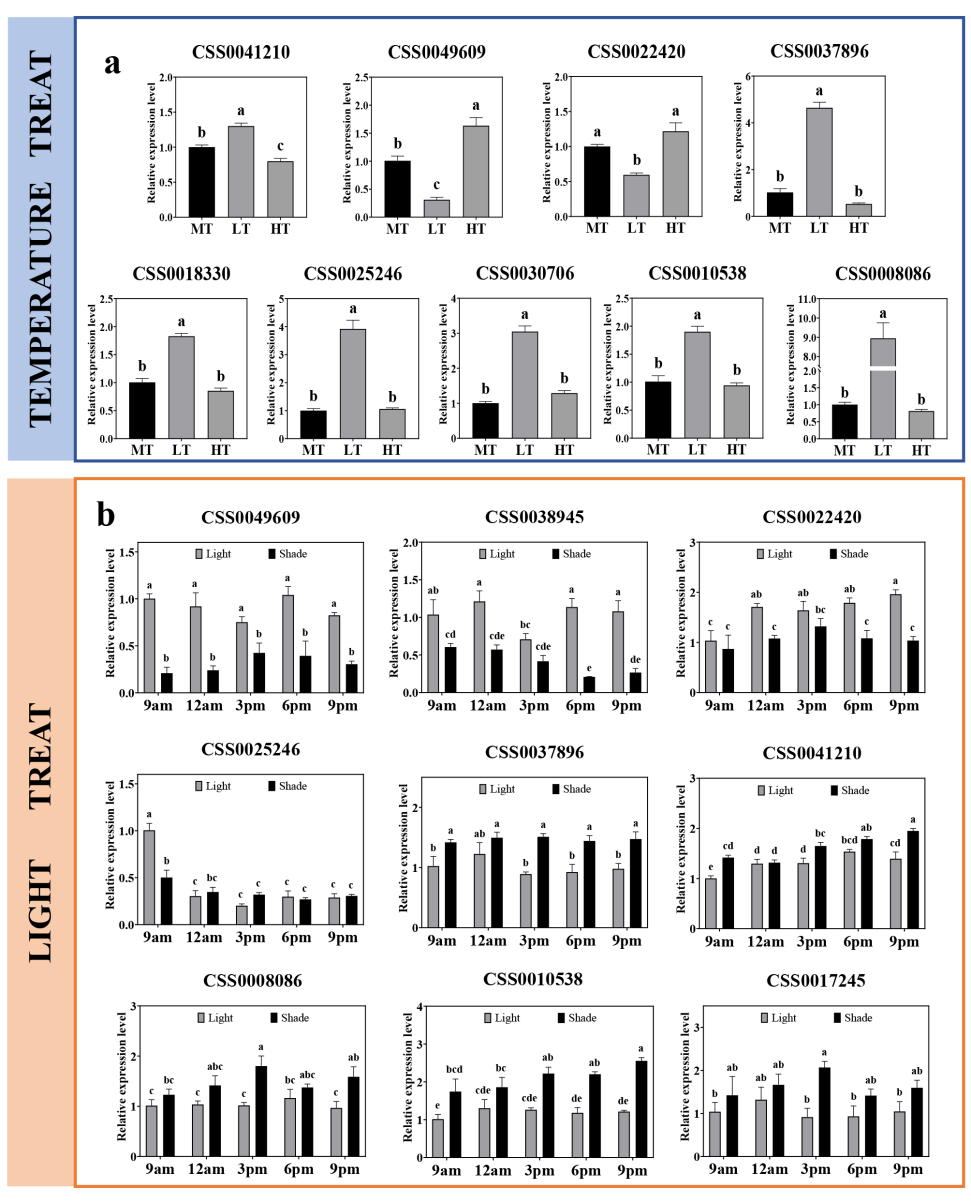
Expression profiles of *CsAP2/ERFs* under treatments. (**a**) Temperature treatment. MT: middle temperature (15 °C), LT: low temperature (4 °C) and HT: high temperature (30 °C). (**b**) Light treatment. The error bars exhibit the means ± SE (n = 3) gained from the three independent biological replicates. The letter represents the significance of the differences (LSD test, *P* < 0.05)

The WGCNA analysis mentioned above found 12 *CsAP2/ERFs* which had the potential relationships with bud break related genes, and the temperature experiments were performed to further verify whether these genes were involved in bud break under temperature-controlled processes. And interestingly, nine *CsAP2/ERFs*, which responded to high (30 °C) or low temperature (4 °C) treatment, were discovered (Fig. 7a). The results of the expression levels indicated that they were all sensitive to low temperature, and CSS0041210 and CSS0049609 were simultaneously influenced by high temperature.

### Expression profiles of ***CsAP2/ERFs*** under light treatment

The expression profiles of 12 *CsAP2/ERFs* screened by WGCNA were detected under light treatment. As the results showed in Fig. 7b, nine *CsAP2/ERFs* responded to light, and four of them were down-regulated under shade treatment while five were up-regulated. CSS0049609 expressed significantly different at five sampling times, however, CSS0025246 and CSS0017245 distinguished at only one time.

## Discussion

The AP2/ERF family is vital in plant development and stress resistance [2]. However, the identification and functional studies of this family remain poorly understood due to the complex genetic background of tea plant. 89 *CsAP2/ERFs* were previously characterized using transcriptome data [26]. In this study, we identified more *CsAP2/ERFs* (178) in the genome of tea plant, and they were grouped into five subfamilies according to the contained domains and the sequence conservation. The number of *AP2/ERFs* greatly varies among different species, as shown in Table 1. The number of *CsAP2/ERFs* in tea plant is relatively large among the plants we have listed. This condition could be caused by the two WGD events in tea plant during the process of genome evolution [39]. In addition, 72 pairs of WGD or segmental duplication were detected in tea plant, making a major construction to the increase in the number of *CsAP2/ERFs*, which is different from the main amplification of other transcription factor families, such as WRKY and PME [40, 41]. The amplification of *AP2/ERFs* in several plants, such as pear [42] and pumpkin [43], is also dominated by segmental duplication. Accordingly, segmental duplication may be a main extension form of *AP2/ERFs*. Moreover, not all duplicated genes had similar expression profiles (Fig. 5), which had also been confirmed in pumpkin [43]. Based on the result of the comparison of the synteny of *AP2/ERFs* between tea plant, Arabidopsis and poplar, more gene pairs are present in tea plant and poplar. Given that both are woody perennials, more similar selective pressure and closer relationship may support the formation of more orthologous genes [39, 44, 45].

The differences in gene structure and composition may contribute to the functional diversity of *AP2/ERFs* [43, 46]. The gene structure analysis showed that the same group or subfamily shared similar gene structures in the *CsAP2/ERF* family. For example, the *CsAP2* subfamily tended to contain three to nine short and tandem CDS regions (Figure S2c), while the *CsDREB* and *CsERF* subfamilies were more likely to form an intron-free structure (Figure S3c, 4c). The structural characteristics of these subfamilies are consistent with the *AP2/ERFs* in other plant [43, 47, 48]. The classification results obtained from phylogenetic trees basically match the prediction results of the conserved domain (Figure S2-S6), indicating that the conserved domains are also an important identification feature of the classification in the *AP2/ERF* family. The analysis of the motif composition revealed that most *CsAP2/ERFs* contained motifs 1, 2 and 4, which were associated with the AP2 domain. Additionally, the subfamilies were differentiated into their unique motifs, such as motif 6 of the *CsDREB* subfamily, motif 14 of the *CsERF* subfamily and motif 15 of the *CsAP2* subfamily, which may support their different functions [49]. Meanwhile, the exogenous hormones and environmental signals may be vital in regulating the transcriptional activity of *AP2/ERFs* [2], and the recognition of these signals needs to be accomplished by *cis*-acting elements. The analysis of the *cis*-acting elements on the promoters of *CsAP2/ERFs* proved that there were various types of hormone response elements and signal-sensing elements (Table S6), such as abscisic acid responsive elements (ABRE), gibberellin responsive elements (TATC-box, P-box and GARE-motif), light responsive elements (ACE) and low temperature responsive elements (LTR). The existence of multiple signal-sensing elements supports the involvement of *AP2/ERFs* in plant physiology and metabolism.

AP2/ERFs are involved in the construction of plant growth and development system and have been confirmed to play a regulatory role in the bud break of woody perennials, such as poplar [20, 22], pear [19] and peach [23]. Most tea plants located in the temperate tea producing area need to experience a cycle of spring bud break and winter dormancy as a member of woody perennials [44, 50, 51]. We observed the bud break process of the tea buds from November 2021 to March 2022 (from S1 to S4) (Figure S1). The tea buds experienced paradormancy, endodormancy and ecodormancy in S1-S3, respectively. The growth of the tea buds stopped at this time due to the surrounding environment or endogenous signals [52, 53]. At the end of S3, the growth points of the tea buds regained their growth capacity but remained in a state of growth arrest due to the limitations of growth conditions [54]. Next, the growing substances in the tea buds continued to accumulate in S4, which made the tea buds enter the expansion period (T10-T12). On March 19, 2022 (T13), the tea buds broke. Consequently, tea plant entered the one and a bud (one bud with one leaf) stage.

Favorable external environment is the key factor for spring bud break of tea plant. Temperature and light are essential in tea plant dormancy to bud break transition[31, 55–59]. Here, we investigated the *cis*-elements of *CsAP2/ERFs* on their promoters and showed that many *cis*-elements were signal perception elements, such as LTR (low temperature response), ACE and G-box (light response). By calculating the correlation between the expressed *CsAP2/ERFs* with daily minimum temperature (DLT), daily maximum temperature (DHT) and daily mean temperature (DMT), it was found that most genes were related to the DLT, accounting for 42.50% (|r| ≥ 0.5) (Table S7). Meanwhile, the temperature and light treatment found that nine *CsAP2/ERFs* could significantly respond to low temperature, and nine could respond to light. Among the above-mentioned genes, seven of them can respond to both low temperature and light.

Tea plant regenerates productive buds in spring under suitable conditions, and this phase is a multi-signal regulation process, which often occurs with altered gene expressions [33, 35, 60, 61]. The expressions of *CsAP2/ERFs* were detected by RNA-seq during the whole process from winter dormancy to spring bud break. The cluster analysis divided the gene expressions into clusters A-F. The gene expressions of clusters A and B were highest at the end of the dormant stage and the tea bud expansion stage. The high-level expression of cluster C-F appeared in the early of stage of dormancy, they expressed barely near the tea bud break. The seasonal expression analysis of woody perennials showed that *AP2/ERFs* had a huge expression transition after chilling accumulation of dormancy or before bud break. In poplar, the expression of *EBB3* was induced by low temperature in the winter/spring months (November to March) [20]. The similar expression pattern of *PpEBB* was found in pear [19]. Compared with poplar and pear, cluster A and B were more synchronized with the process of tea plant bud break in spring. Further research revealed that CSS0047280 in cluster A is the orthologous gene of *ESR2* in Arabidopsis. *ESR2* is vital in shoot regeneration through the transcriptional regulation of *CUC1*, and ectopic expression of *CUC1* could promote adventitious shoot formation from Calli through Shoot Apical Meristem (SAM) activation [62]. This work provided a reference for studying the potential mechanism of CSS0047280 in tea plant bud break. In addition, the CSS0035725 in cluster B had high homology with *EBB3*, the bud break regulation gene in poplar [20]. This result also indicates that this gene may have a similar function to *EBB3* in regulating the bud break. Furthermore, the genes in other clusters were down-regulated before tea plant bud break, showing an opposite expression pattern to the genes in clusters A and B which also suggested a possible negative regulatory mechanism.

D-type cyclins are an important cell cycle progression checkpoint, whose expression correlates with bud reactivation of growth at the bud break, and participates in compound pathways in the regulation of bud break in woody perennials [20, 34, 63, 64]. In poplar, *CYCD3.1* promotes poplar bud break, and it is up-regulated by *EBB3* which is up-regulated by *EBB1*. The entire pathway is induced by low temperature signals [20]. Our study found a *CsAP2/ERF* (CSS0049609) that could respond to low temperature signal and were highly correlated with D-type cyclin genes in expression. CSS0039817, the orthologous gene of poplar *CYCD3.1*, had a similar expression pattern to CSS00033589 and CSS0049609 in module Brown. The correlation coefficients of gene expression were 0.89 (CSS0039817 and CSS0033589) and 0.90 (CSS0039817 and CSS0049609) during spring bud break (Table S9). These results demonstrate that CSS00033589, CSS0049609 and CSS0039817 may be similar to the regulation mechanism of *EBB1* and *EBB3* on *CYCD3.1*. Moreover, CSS0028484 and CSS0022420 had high sequence similarity and similar expression profile with CsAIL, an AP2/ERF transcription factor reported in tea plant [34]. Previous studies have shown that *CsAIL* may be an upstream regulatory gene of tea plant D-type cyclin genes *CsCYCD3.2* and *CsCYCD6.1*, which is consistent with our experimental results. The above-mentioned results provide evidence to prove that the *CsAP2/ERFs* are involved in the expression and regulation of D-type cyclin genes, thereby affecting tea plant bud break.

## Conclusions

This study performed a systematic analysis of the CsAP2/ERF family in tea plant. A total of 178 *CsAP2/ERFs* were identified and divided into five subfamilies. The evolution, gene location, conserved motifs, and *cis*-acting element features of *CsAP2/ERFs* were investigated. Furthermore, the expression patterns of *CsAP2/ERFs* in different periods of tea plant bud break were analyzed. Nine low temperature responsive and nine light responsive *CsAP2/ERFs* were found during the experiment of the temperature and light treatments. Finally, *CsAP2/ERF*s may be an upstream regulator of D-type cyclin genes, which affected tea plant bud break in spring. Our study provided a new direction for further research on the functioning of *CsAP2/ERFs* in tea plant bud break.

## Materials and methods

### Identifications of the ***CsAP2/ERFs***

The genome data and annotation information of the chromosome-level reference of tea plant were downloaded from TPIA (http://tpdb.shengxin.ren/) [65]. The Hidden Markov Model (HMM) file was downloaded from InterPro (https://www.ebi.ac.uk/interpro/download/Pfam/) [66] and submitted to Simple HMM Search of TBtools [67] along with the AP2 domain ID (PF00847). The above-mentioned steps were used to retrieve the AP2/ERF proteins from the tea plant genome [39]. After eliminating repetitive sequences, the rest of the proteins were analyzed by CD-search of NCBI (https://www.ncbi.nlm.nih.gov/Structure/cdd/wrpsb.cgi) [68]. Ultimately, 178 *CsAP2/ERFs* were identified. The ExPAsy ProtParam (https://web.expasy.org/protparam/) [69] was employed to predict the physical and chemical parameters of the CsAP2/ERF proteins, including the amino acids, molecular weight and isoelectric points (Table S1).

### Phylogenetic tree construction

The protein sequences of 178 *CsAP2/ERFs* were extracted from the tea plant genome by TBtools [67], and the AP2/ERF protein sequences of Arabidopsis were downloaded from the PlantTFDB (http://planttfdb.gao-lab.org/) [70]. The conserved domains were predicted through CDD (http://www.ncbi.nl.nih.gov/Structure/cdd/wrpsb.cgi) [71], and the predicted results were downloaded for phylogenetic analysis. Then, all sequences were aligned by MUSCLE in MEGA [72] with the neighbor-joining (NJ) method using the Poisson model, and the bootstrap test was replicated 1000 times. The result was imported into the iTOL (https://itol.embl.de/) [73] to display the phylogenetic tree.

### Chromosomal distribution and gene duplication

The location information of *CsAP2/ERFs* on the chromosomes was collected by the TeaGVD (http://www.teaplant.top/teagvd) [74]. One Step MCScanX of TBtools [67, 72] was used to identify the synteny regions on the tea plant genome [39] based on E-value ≤ 1E −5. Then, the paralogous relationships were extracted by the ID information of *CsAP2/ERFs*. The genome data and annotation information of the Arabidopsis and poplar were downloaded from EnsemblPlants (http://plants.ensembl.org/info/data/ftp/index.html) [75]. The above-mentioned files were used for synteny analysis together with the genome information of the tea plant [39]. The chromosomal localization and gene synteny were visualized by TBtools [67]. Finally, non-synonymous substitutions (Ka) and synonymous substitutions (Ks) of the paralogous genes were calculated using KaKs_Calculator 2.0 with the calculation method NG [76].

### Gene structure, conserved motif and promoter analysis

The structures (CDS, UTR and introns) of 178 *CsAP2/ERFs* were extracted from the genome annotation information of tea plant [39] and displayed through the GSDS (http://gsds.gao-lab.org/) [77]. Then, the TBtools [67] was used to extract the genome sequences of 178 *CsAP2/ERFs* and the 2 kb sequences upstream from the transcription start site. The MEME (https://meme-suite.org/meme/tools/meme) [78] was used to identify conserved motifs. The 2 kb sequences upstream of the genes were analyzed by the PlantCARE (http://bioinformatics.psb.ugent.be/webtools/plantcare/html/) [79] for predicting the *cis*-acting elements in the promoters of *CsAP2/ERFs*.

### Plant growth and treatment

The 8-year-old early-sprouting tea cultivar ‘Longjing43’ was grown in Shengzhou experimental base, Tea Research Institute of Chinese Academy of Agricultural Sciences (TRICAAS), Zhejiang, China. From November 1, 2021 to March 19, 2022, the apical buds were sampled 13 times depending on the development of buds and weather conditions. The records of sampling can be obtained from Figure S1 and Table S3. In the same field in Shengzhou, tea plants with consistent growth were selected for light treatment. The tea plant in light group grew naturally without additional treatment. the shade group was shaded for four days before sampling, and the shading rate was 95%. On the fifth day, two groups of tea plant were sampled every three hours separately, from 9am to 9pm on December 14, 2021. Potted early-sprouting tea cultivar ‘Longjing43’ (2-year-old) with consistent and robust growth was cultivated in the greenhouse. The tea plant was then moved into the high (30 °C), low (4 °C) and middle (15 °C) temperature climate chambers for treatment. The humidity of the artificial climate chamber used for temperature treatment was set at 70%, and the photoperiod of 14 h of light (10,000 lx) and 10 h of darkness is maintained. After two days of treatment, the apical buds were harvested, and frozen in liquid nitrogen and stored at −80 °C. Three biological replicates were set up for each sample.

### Gene expression analysis based on RNA-seq and qRT-PCR

The total RNA was extracted by EASY-spin Plus Complex Plant RNA Kit (Aidlab Biotechnologies Company, Beijing, China) and used to construct cDNA libraries. The Agilent bioanalyzer 2100 system was used to detect the library quality. The qualified libraries were sequenced on the NovaSeq 6000 platform (Illumina Inc., CA, USA) developed by the Novogene Bioinformatics Technology Co., Ltd (Beijing, China). After removing the unqualify reads (adapter reads, poly-N reads and low-quality reads), the clean reads were aligned to the tea plant genome using HISAT2 [80]. The average total reads were 44,917,372, and the average map rate up to 85.94%. The transcript expression levels of individual genes were quantified using FPKM values, which were counted by featureCounts basing on the length of the gene and reads count mapped to the genes.

Subsequently, the gene expression heatmap was generated by TBtools [67]. Real-time qPCR was conducted on LightCycler® 480 II (Roche Molecular Biochemicals, Mannheim, BW, Germany) using LightCycler® 480 SYBR® Green I Master (Roche Molecular Biochemicals, Mannheim, BW, Germany). Each treatment performed three biological and three technical replicates. The relative expression level was calculated by using the 2^−ΔΔCT^ method [81]. The internal reference gene was *CsGAPDH*. The primer sequences of the reference genes and *CsAP2/ERFs* are listed in Table S2.

### WGCNA analysis

WGCNA [82] was performed by the R package. The gene expression data were obtained from RNA-seq. All genes were filtered by the standard of FPKM > 1, and we set the soft threshold to nine to construct the network. The dissimilarity between genes was used for the hierarchical clustering of genes, and a hierarchical clustering tree was established. Then, the tree was cut into 15 modules (the minimum number of genes in the module was 30) by using the dynamic shearing method, and the modules with a coefficient of dissimilarity less than 0.25 were merged. The WGCNA results were used to identify gene sets with high covariation and to mine potential interacting genes. The gene co-expression network was visualized using Cytoscape [83].

## Electronic supplementary material

Below is the link to the electronic supplementary material.

Additional file 1: Supplemental tables

Additional file 2: Supplemental figures

## Data Availability

The datasets presented for this study can be found in the Supplementary Materials and NCBI with the accession number PRJNA898859. The direct link for the NCBI database is https://www.ncbi.nlm.nih.gov/search/all/?term=PRJNA898859.

## Abbreviations

AP2/ERF: APETALA2/Ethylene responsive factor
*WGCNA*: weighted correlation network analysis
*DREB*: dehydration responsive element binding protein
*RAV*: Related to ABSCISIC ACID INSENSITIVE3 (ABI3)/VIVIPAROUS1(VP1)
WGD: whole genome duplication
*FPKM*: fragments per kilobase of transcript per million mapped reads
