# Supplementary material for: Comprehensive analysis and expression profiles of the AP2/ERF gene family during spring bud break in tea plant (Camellia sinensis)
Source: BMC Plant Biol. 2023 Apr 20;23:206. doi: 10.1186/s12870-023-04221-y (PMC10116778; doi:10.1186/s12870-023-04221-y)
Supplement: Supplementary file 2 — Additional file 2: Supplemental figures [file 12870_2023_4221_MOESM2_ESM.docx]

# Additional file 2


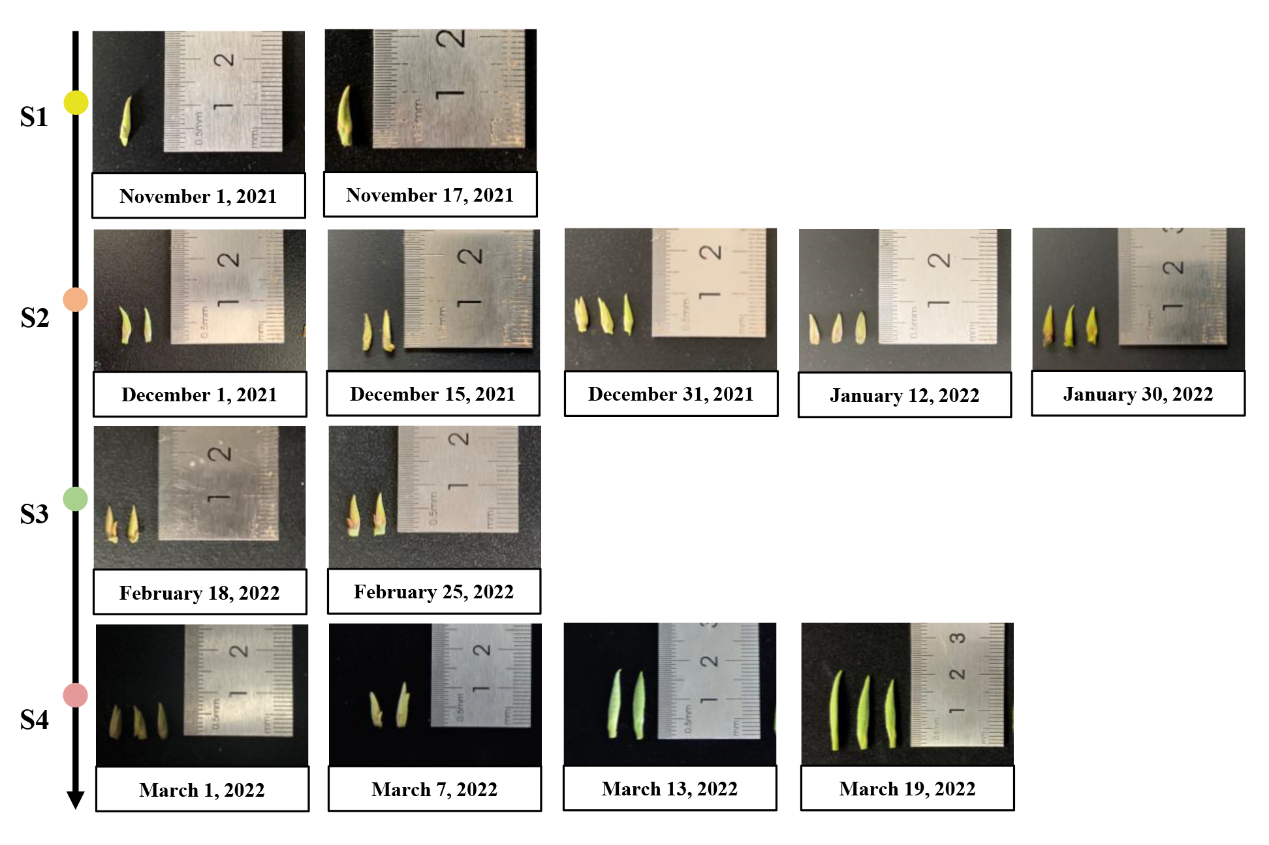


**Figure S1.** The records of sampling.


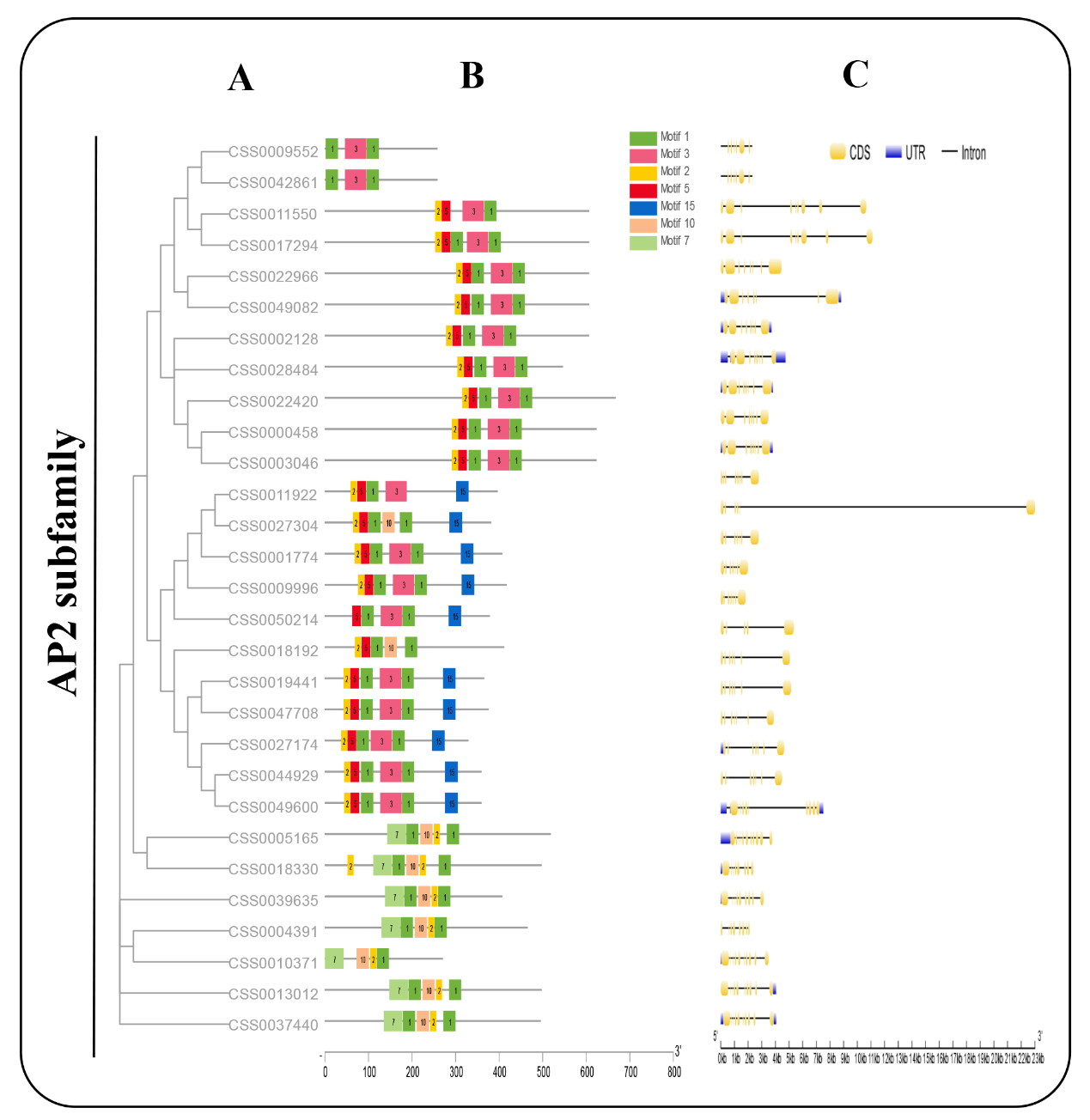


**Figure S2.** Phylogenetic tree (A), conserved motif (B) and gene structure (C) of the *CsAP2s*.


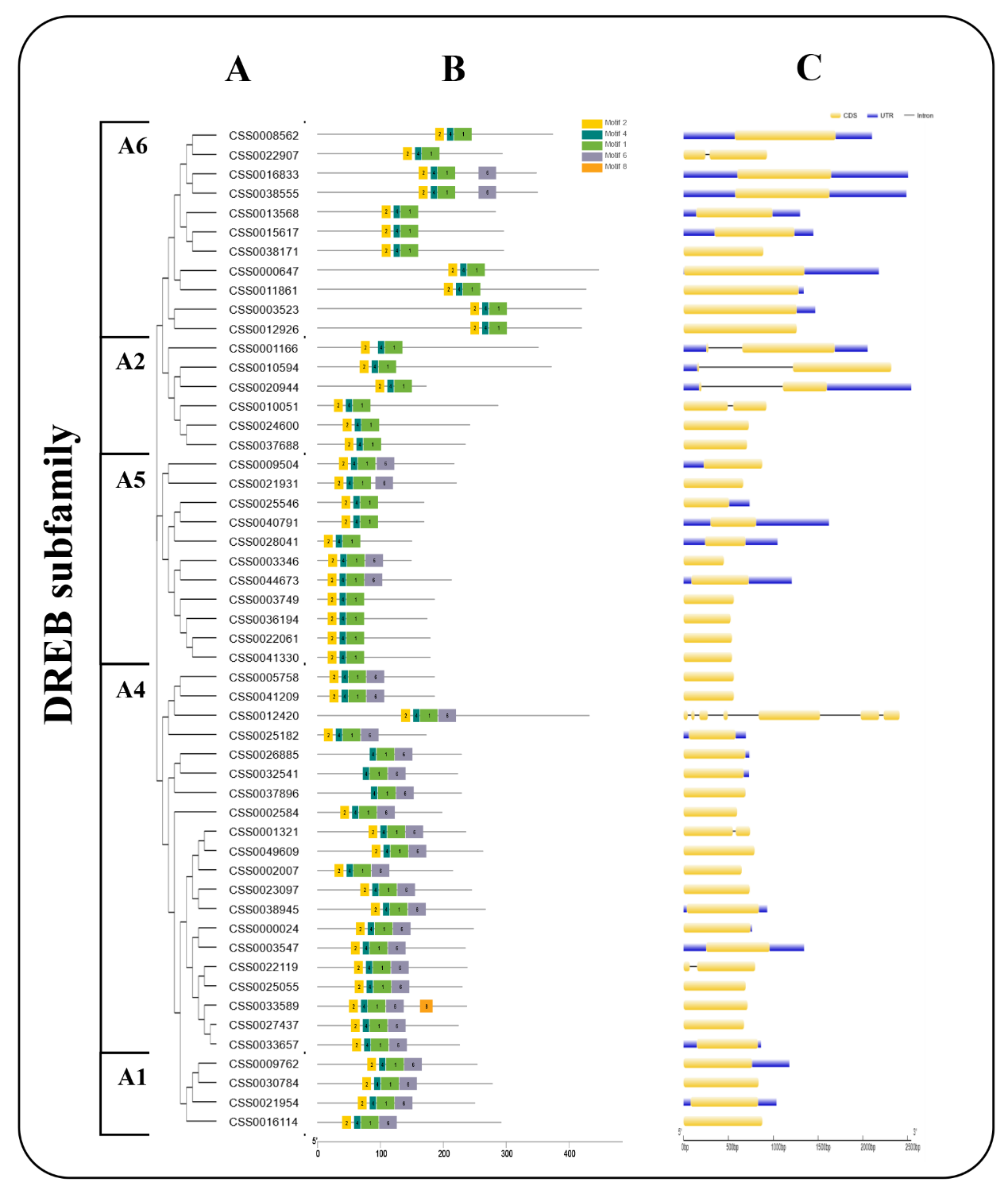


**Figure S3.** Phylogenetic tree (A), conserved motif (B) and gene structure (C) of the *CsDREBs*.


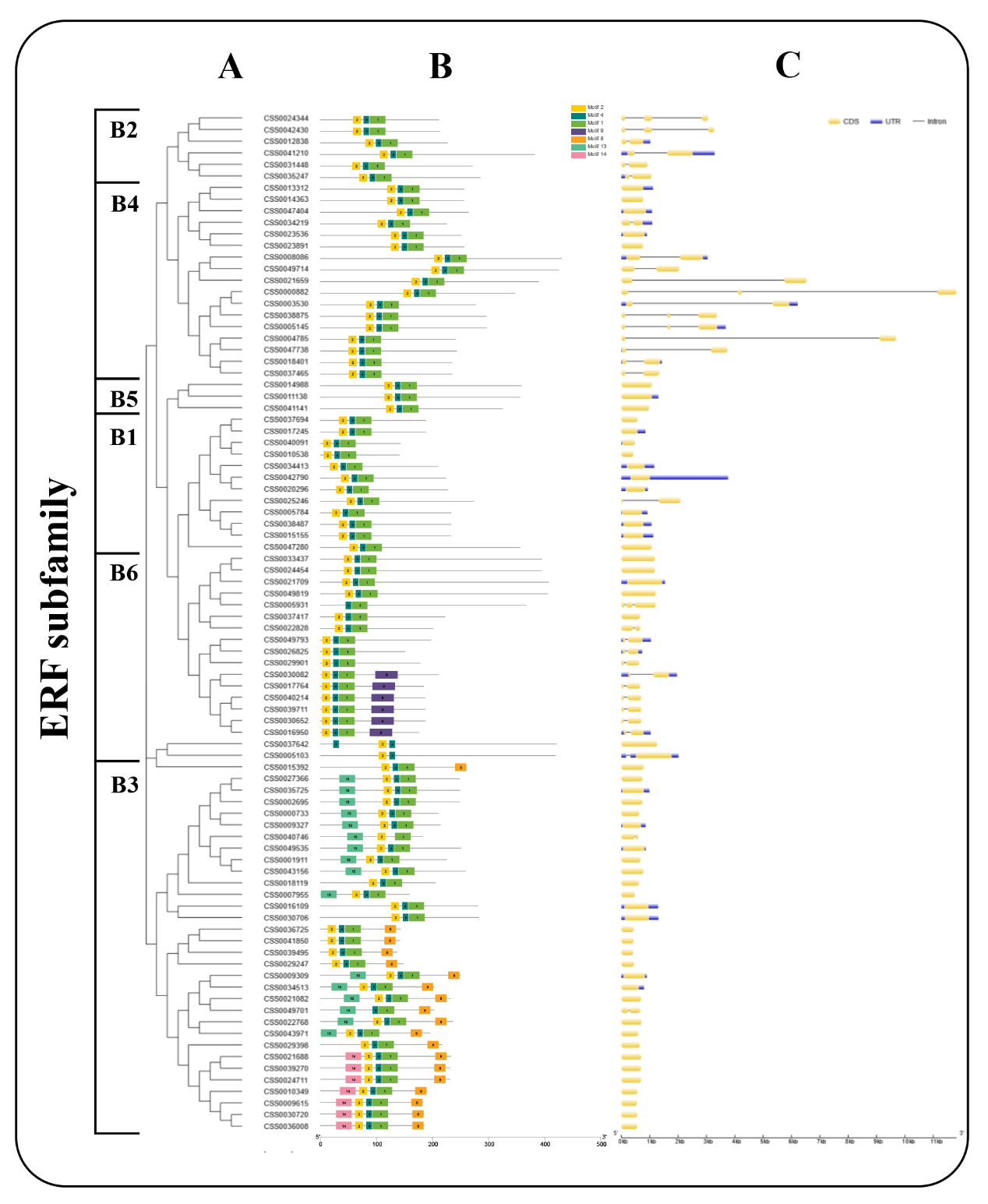


**Figure S4.** Phylogenetic tree (A), conserved motif (B) and gene structure (C) of the *CsERFs*.


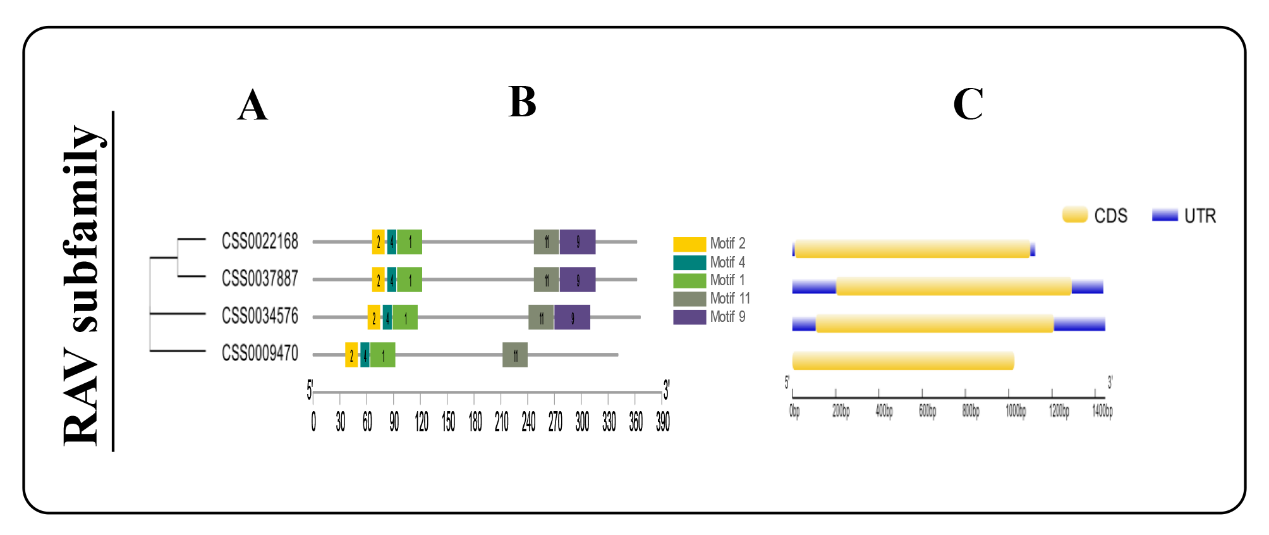


**Figure S5.** Phylogenetic tree (A), conserved motif (B) and gene structure (C) of the *CsRAVs*.


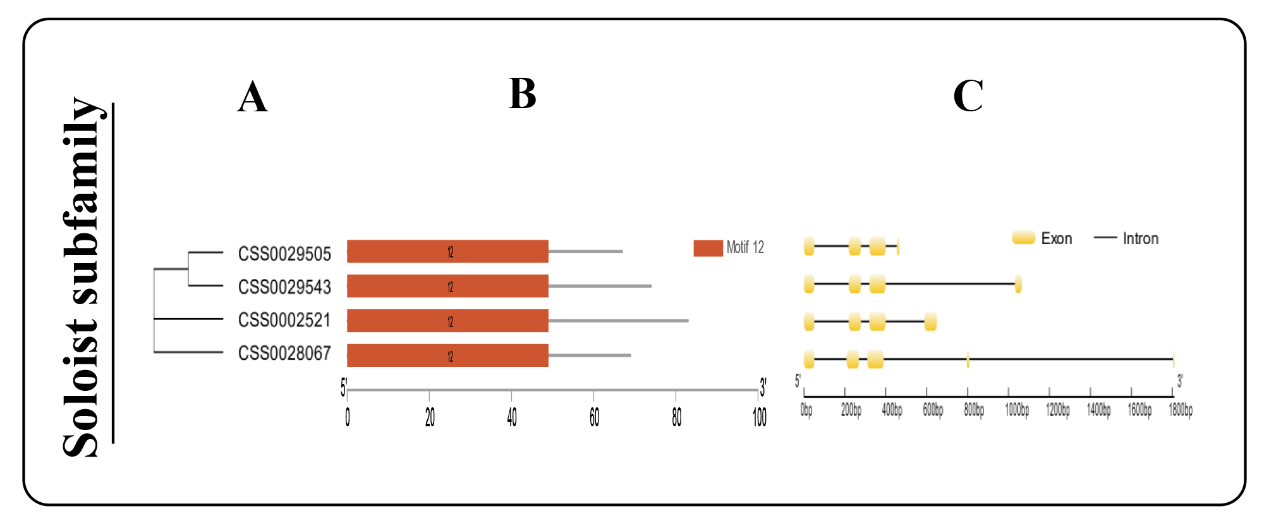


**Figure S6.** Phylogenetic tree (A), conserved motif (B) and gene structure (C) of the *CsSoloists*.
